# Immunogenicity mechanism of mRNA vaccines and their limitations in promoting adaptive protection against SARS-CoV-2

**DOI:** 10.7717/peerj.13083

**Published:** 2022-03-09

**Authors:** Mohd Zulkifli Salleh, Mohd Nor Norazmi, Zakuan Zainy Deris

**Affiliations:** 1 Department of Medical Microbiology & Parasitology, School of Medical Sciences, Universiti Sains Malaysia, Kota Bahru, Kelantan, Malaysia; 2 School of Health Sciences, Universiti Sains Malaysia, Kota Bahru, Kelantan, Malaysia

**Keywords:** mRNA vaccine, COVID-19, SARS-CoV-2, Innate immune response, Adaptive immune response, Humoral immune response, Cell-mediated immune response, Immunogenicity mechanism, Variants of concern

## Abstract

Since the emergence of severe acute respiratory syndrome coronavirus 2 (SARS-CoV-2), the causative agent of coronavirus disease 2019 (COVID-19) in late 2019, hundreds of millions of people have been infected worldwide. There have been unprecedented efforts in acquiring effective vaccines to confer protection against the disease. mRNA vaccines have emerged as promising alternatives to conventional vaccines due to their high potency with the capacity for rapid development and low manufacturing costs. In this review, we summarize the currently available vaccines against SARS-CoV-2 in development, with the focus on the concepts of mRNA vaccines, their antigen selection, delivery and optimization to increase the immunostimulatory capability of mRNA as well as its stability and translatability. We also discuss the host immune responses to the SARS-CoV-2 infection and expound in detail, the adaptive immune response upon immunization with mRNA vaccines, in which high levels of spike-specific IgG and neutralizing antibodies were detected after two-dose vaccination. mRNA vaccines have been shown to induce a robust CD8^+^T cell response, with a balanced CD4^+^ T_H_1/T_H_2 response. We further discuss the challenges and limitations of COVID-19 mRNA vaccines, where newly emerging variants of SARS-CoV-2 may render currently deployed vaccines less effective. Imbalanced and inappropriate inflammatory responses, resulting from hyper-activation of pro-inflammatory cytokines, which may lead to vaccine-associated enhanced respiratory disease (VAERD) and rare cases of myocarditis and pericarditis also are discussed.

## Introduction

The ongoing global transmission of coronavirus disease 2019 (COVID-19) is being caused by severe acute respiratory syndrome coronavirus 2 (SARS-CoV-2), a positive-strand single-stranded (ss) RNA virus, which has infected more than 375 million people and claimed the lives of at least 5.6 million people, worldwide (as of January 2022, WHO). It is a global phenomenon that has caused significant social, economic and political impacts and challenges worldwide, leading to travel restrictions and severely disrupting public health services. SARS-CoV-2 typically induces respiratory-like illnesses that develops into pneumonia and symptoms of the disease are mild to severe, which include cough, sore throat, fever or chills, headache, breathing difficulties, loss of taste or smell, nausea and diarrhea (CDC, 2021). Similarly, severe acute respiratory syndrome coronavirus (SARS-CoV) and Middle East respiratory syndrome coronavirus (MERS-CoV) are the other two pathogenic and highly transmissible coronaviruses that have caused major deadly pneumonic pandemics in humans since the 21st century. The betacoronavirus SARS-CoV-2 belongs to the subfamily *Coronavirinae* of the family *Coronaviridae* and the order *Nidovirales*, and contains a large genome of about 30,000 bases (Chen et al., 2020b). The coronavirus is composed of an envelope that contains envelope (E) and membrane (M) proteins, coated with homotrimeric spike (S) glycoproteins, and a helical capsid consisting of nucleocapsid (N) proteins bound to the RNA genome. The full-length SARS-CoV-2 S glycoprotein consists of two subunits, namely S1 and S2 subunits. Viral entry into host cells is mediated by the S glycoprotein binding to its receptor, angiotensin-converting enzyme 2 (ACE2) through the receptor-binding domain (RBD) of the S1 subunit. The binding subsequently induces viral fusion to the host cellular membranes through irreversible conformational changes, mediated by its S2 subunit (Hoffmann et al., 2020). The full-length S glycoprotein, S1 subunit and RBD have been shown to induce neutralizing antibodies (nAbs) and T cell-mediated immunity, and serve as promising targets for vaccine development against SARS-CoV-2 (Amanat & Krammer, 2020). On the verge of the ongoing calamity and death, the urgent need for effective vaccines providing protection against the virus is crucial. In this review, we discuss the recent development of vaccines for COVID-19, with the focus on mRNA vaccines and its immunogenicity mechanism and limitations in promoting adaptive protection against SARS-CoV-2.

## Survey Methodology

In order to extensively review the literature on immunogenicity mechanism of mRNA vaccines against SARS-CoV-2, we conducted thorough searches in PubMed and Google Scholar using the following terms: vaccine development; spike; mRNA optimization; lipid nanoparticle; adjuvants; immune response; innate immune response; adaptive immune response; humoral immune response; cell-mediated immune response; cellular immune response; antibody; IgA; IgM; IgG; cytokines; T cell; B cell; or macrophages in combination with SARS-CoV-2 or COVID-19 or Pfizer vaccine or BNT162b2 or Moderna vaccine or mRNA-1273. While current literatures published on mRNA vaccine/SARS-CoV-2/COVID-19 in English within the last two years were considered, literatures that contained relevant critical information to the review’s objectives were also included. This information will be valuable for scientists and researchers in guiding the development of mRNA vaccines for protection against the ongoing COVID-19 pandemic.

## The Other Covid-19 Vaccines

**Figure 1:**
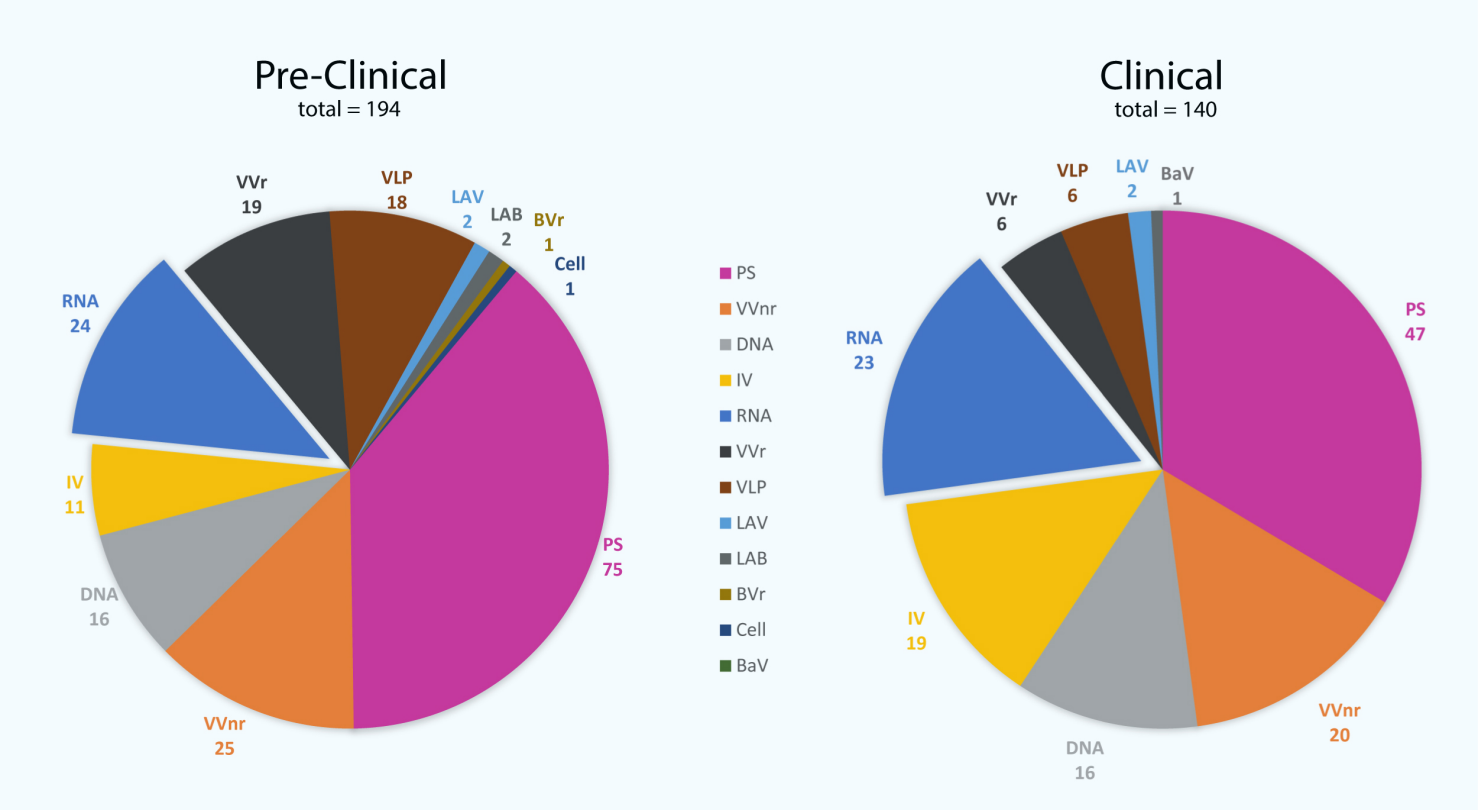
The number of vaccine candidates being developed against severe acute respiratory syndrome coronavirus 2 (SARS-CoV-2) to date. There are 140 vaccine candidates that are currently at least in stage 1 clinical trials, whereas 194 vaccines are in pre-clinical stages. PS, protein subunit; VVnr, viral vector (non-replicating); DNA, DNA-based vaccines; IV, inactivated virus; RNA, RNA-based vaccines; VVr, viral vector (replicating); VLP, virus-like particle; LAV, live-attenuated virus; LAB, live-attenuated bacterial vector; BVr, bacterial vector (replicating); Cell, cellular-based vaccine; BaV, bacterial antigen-spore expression vector (WHO, 2022).

At this time in the pandemic and in the absence of effective therapeutic treatments for COVID-19 patients, a vaccine is the most promising way to control the pandemic and stop the spread of SARS-CoV-2. There have been unprecedented efforts in acquiring effective vaccines against the disease. To date, there are 334 vaccine candidates in development worldwide (Fig. 1), in which 140 vaccines are in clinical and 194 vaccines are in pre-clinical developments (as of 24th January 2022) (WHO, 2022). Vaccines provide adaptive immunity against the disease and are generally made of an inactivated or live attenuated pathogen, as well as recombinant protein subunits, vectored and conjugated vaccines, and virus-like particles. Inactivated vaccines are developed using the SARS-CoV-2 virus growing in cell culture, typically Vero cells (Gao et al., 2020; Wang et al., 2020). The cultured virus is then inactivated using heat or chemicals such as β-propiolactone or formaldehyde to inhibit the viral reproduction capability, while maintaining its immune response stimulation. Examples of inactivated vaccines include CoronaVac (previously known as PiCoVacc), which is developed by the Chinese company Sinovac Biotech (Gao et al., 2020), BBIBP-CorV and WIBP-CorV by Sinopharm (Wang et al., 2020), BBV152 (also known as Covaxin) by the Indian company Bharat Biotech (Ella et al., 2021), CoviVac by the Russian Academy of Sciences and VLA2001 by the French company Valneva SE. These vaccines may contain adjuvants such as alum (aluminum hydroxide), alhydroxiquim-II, CpG 1018 and others to induce high levels of antibody production and long-lasting immunity.

Live attenuated vaccines are manufactured by using a pathogen with reduced virulence that replicates to a limited extent, which does not cause disease but retains the ability to stimulate immune response. Attenuation can be achieved by physical or chemical approaches, as well as genetic modifications using codon de-optimization or gene deletion. To date, only two live attenuated vaccine candidates have entered Phase 1 clinical trial (Fig. 1): one that is being developed by Codagenix, in collaboration with the Serum Institute of India (codenamed as COVI-VAC), and the other is MV-014-212, manufactured by Meissa Vaccines (WHO, 2022). Both vaccines are formulated to be administered as a single intranasal dose, developed using codon de-optimization technology. A pre-clinical safety and efficacy report published in July 2021 showed that the COVI-VAC vaccine protected hamsters from COVID-19 and promoted the formation of a potent virus-neutralizing antibody response (Wang et al., 2021b). Two other codon de-optimized live attenuated COVID-19 vaccine candidates in pre-clinical stage are being developed by Mehmet Ali Aydinlar University/Acıbadem Labmed Health Services A.S. and Indian Immunologicals Ltd/Griffith University (WHO, 2022).

A large number of SARS-CoV-2 vaccine candidates that are currently being developed rely on viral vectors, which carry a relevant gene from the target virus, the S gene for SARS-CoV-2 for example. The SARS-CoV-2 viral vector vaccines are based either on replicating or inactivated non-replicating viral vector platforms, typically adenovirus although other viral platforms such as modified lentivirus, vaccinia Ankara, Newcastle disease virus, Sendai virus, influenza, measles as well as rabies viruses are also being used. There are 6 replicating and 20 non-replicating viral vector vaccine candidates in clinical development of at least in Phase 1 trial (Fig. 1), with the Oxford/AstraZeneca AZD1222 (ChAdOx1-S, commercialized as Covishield or Vaxzevria), the CanSino AD5-nCOV (commercialized as Convidecia) and the Janssen/Johnson & Johnson Ad26.COV2.S are the only non-replicating viral vector vaccines that have passed the Phase 4 clinical trial and currently being distributed for mass vaccination (WHO, 2022). The Oxford/AstraZeneca vaccine is given by intramuscular injection, two doses administered four weeks apart and using the modified chimpanzee adenovirus ChAdOx1 as a vector. A safety report published in March 2021 showed an efficacy of 76.0% from participants who received a single standard dose from day 22 to day 90 of vaccination, whereas in participants who received two standard doses, the efficacy was increased to 81.3% (Voysey et al., 2021). The CanSino AD5-nCOV vaccine is given by intramuscular injection, aerosol inhalation, or both and has been shown to induce higher nAbs percentage in individuals with history of COVID-19 infection than those without prior COVID-19 infection (Hernández-Bello et al., 2021). Aerosolised Ad5-nCoV is well received, although two doses of aerosolised AD5-nCoV elicited similar nAb responses to one dose of intramuscular injection (Wu et al., 2021). A single dose of the Janssen/Johnson & Johnson Ad26.COV2.S vaccine, on the other hand, induced rapid binding and nAb responses as well as robust CD4^+^ and CD8^+^ T-cell mediated immune responses (Stephenson et al., 2021). The vaccine has been shown to protect against symptomatic and asymptomatic SARS-CoV-2 infection, and is effective against severe disease, including hospitalization and death (Sadoff et al., 2021).

Recombinant protein subunit vaccines, in which viral proteins such as the whole S glycoprotein or the recombinant RBD of the S glycoprotein, are injected intramuscularly, have the potential to elicit protection to the host against the viral infection. However, due to the limited number of viral components involved, their effectiveness in protecting the host is limited because they do not display the complete antigenic complexity of the virus. Adjuvants are therefore needed to enhance the stimulatory capacity of subunit vaccines. For instance, full length recombinant SARS-CoV-2 S glycoprotein nanoparticle adjuvanted with Matrix-M1 is used in the production of the Novavax NVX-CoV2373 vaccine (Keech et al., 2020). In addition, two Sanofi/GSK COVID-19 vaccines, code-named VAT0002 and VAT0008 (commercialized as Vidprevtyn) are adjuvanted recombinant S protein subunit vaccines and have been reported to show strong immune responses. Preliminary results from a clinical trial of the VAT0002 vaccine as booster showed nAbs increased 9- to 43-fold, regardless of the primary vaccine received (Sanofi, 2021). The full S protein is relatively difficult to express, which contributes to low production rate and high production cost. The RBD of the S protein, although is much smaller, is easier to express and could be produced at large quantities using different expression systems such as insect cells, mammalian cells, yeast and plants, as well as in *Escherichia coli* (Chen et al., 2005; Krammer, 2020). Recently, a RBD-based recombinant protein vaccine, the HIPRA PHH-1 V vaccine, which consists of a novel RBD fusion heterodimer containing the Alpha (B.1.1.7) and Beta (B.1.351) variants of SARS-CoV-2 has been developed. The vaccine showed promising results in protecting mice from COVID-19 infection, with robust activation of CD4^+^ and CD8^+^ T cells (Barreiro et al., 2021). Although many potent nAbs bind to RBD (Ju et al., 2020; Yang et al., 2020), other neutralizing epitopes that are present on the full-length S glycoprotein are absent in the RBD-derived vaccine, which may render the vaccine more prone to impact from antigenic drift and lead to reduced effectiveness.

Recently, the first plasmid DNA vaccine against SARS-CoV-2 was rolled out. The ZyCoV-D vaccine, which is developed by Indian pharmaceutical company Cadila Healthcare, carrying the gene encoding the S glycoprotein and is given by intradermal injection, three doses administered 28 days apart (Dey et al., 2021). The vaccine has been issued with an Emergency Use Authorization (EUA) for use in India. A safety and immunogenicity report published recently showed that the DNA vaccine induced robust humoral and cellular immune responses against the SARS-CoV-2 S protein (Momin et al., 2021). Neutralizing geometric mean titers (GMTs) of immunoglobulin G (IgG) have been shown to increase significantly from 7.00 on day 0 to 1,019.61 on day 70 (14 days after three doses of the vaccine). However, on day 84 (28 days after three doses of the vaccine), GMTs decreased and remained stable at 748.46 (Momin et al., 2021). A double-blind, placebo-controlled Phase III study is currently ongoing and the interim results show that the efficacy of the vaccine to be 67% against symptomatic COVID-19 (Mallapaty, 2021).

## mRNA vaccines against SARS-COV-2

mRNA vaccines have emerged as promising alternatives to conventional vaccines. Although conventional vaccine approaches, such as inactivated and live attenuated pathogens and subunit vaccines provide long and durable protection against dangerous diseases, the need for more rapid development with low-cost manufacture and large-scale deployment become major hurdles to the vaccine development, especially to cater for pandemics. mRNA vaccine provides high potency against a disease with the capacity for rapid development and low-cost manufacturing, providing new opportunity for protection against a variety of emerging dangerous diseases (Pardi et al., 2018). Additionally, conventional vaccines may not suitable for non-infectious diseases, such as cancer. mRNA vaccine encoding tumor-associated antigens with the RNActive technology is currently being evaluated in several clinical trials and may provide a new treatment for cancer (Beck et al., 2021; Fiedler et al., 2016). Cancer vaccination involves the induction of tumor-specific B- and T-cell responses, and has been shown to induce a balanced humoral and cellular immune responses in animal models (Fiedler et al., 2016). Furthermore, mRNA vaccines have been shown to elicit potent immunity against viral diseases in animal models of Ebola, Zika, influenza, rabies, cytomegalovirus and others, using lipid-encapsulated or naked forms of sequence-optimized mRNA (Pardi et al., 2018).

The COVID-19 pandemic has triggered an urgent need for vaccines to be developed as fast as possible against SARS-CoV-2. mRNA vaccines, which can be produced at a faster rate provided new alternative approaches in the protection against SARS-CoV-2. These vaccines are usually administered intramuscularly and directed into human cells, where transcription of the nucleic acid into viral proteins takes place. Currently, there are 23 RNA-based vaccine candidates recorded in the clinical development (Fig. 1) (WHO, 2022). BNT162b2 (Tozinameran), the very first vaccine approved by the Food and Drug Administration (FDA) against SARS-CoV-2, is a lipid nanoparticle-formulated, nucleoside-modified mRNA vaccine, developed by Pfizer Inc. and BioNTech SE. The vaccine, which has been issued with an EUA on 11 December 2020, consists of 2 doses of 30 µg (300 µl each), administered intramuscularly three weeks apart (Lamb, 2021). Based on clinical trials in people aged 16 years and older, the vaccine has been shown to have an efficacy of 95% at protecting against COVID-19 in people without evidence of previous infection (Oliver et al., 2020; Polack et al., 2020). mRNA-1273 (Moderna COVID-19 vaccine) is another nucleoside-modified mRNA vaccine, developed by the United States National Institute of Allergy and Infectious Diseases (NIAID), the Biomedical Advanced Research and Development Authority (BARDA) and Moderna, Inc, which has been issued with an EUA on 18 December 2020. The vaccine is administered by intramuscular injection, two 0.5 ml doses (100 µg) given four weeks apart. The mRNA-1273 has been shown to have an efficacy of approximately 94% at preventing COVID-19 illness, including severe disease, starting 14 days after the first dose (Baden et al., 2021). The development for both vaccines began in January 2020 and in just less than three months later, both vaccines entered Phase I clinical trial. By December 2020, the vaccines were issued with an EUA and are currently being distributed worldwide. Both mRNA-1273 and BNT162b2 carry a piece of mRNA that encodes the SARS-CoV-2 S glycoprotein, the immunogen in which the immune response is activated against the virus upon its expression. BNT162b2 and mRNA-1273 encode the full-length S protein, modified by two proline mutations (S-2P) to maintain it in the prefusion conformation (Corbett et al., 2020a; Walsh et al., 2020).

### Spike glycoprotein

The S protein forms a homotrimeric complex protruding from the viral surface and consists of two functional subunits, S1 and S2, which are responsible for host cell receptor binding and viral membrane fusion, respectively. The smaller S1 subunit comprises an N-terminal domain (NTD) and three C-terminal domains (CTD1–3). CTD1 forms the RBD and contributes to stabilization of the membrane-anchored S2 subunit. The RBD mediates the binding to the host cell receptor, ACE2. The larger S2 subunit contains the machinery for the membrane fusion and consists of a hydrophobic fusion peptide (FP), heptad repeat 1 (HR1), central helix (CH), connector domain (CD), heptad repeat 2 (HR2), transmembrane domain (TM) and cytoplasmic tail (CT) (Kalathiya et al., 2020; Kirchdoerfer et al., 2016; Walls et al., 2020). In all coronaviruses, the S glycoprotein is cleaved upon the binding by host proteases at the S1/S2 junction; cleavage has been suggested to activate the protein for host membrane fusion through irreversible conformational changes, driving the S protein from a pre-fusion to a post-fusion conformations (Cai et al., 2020; Song et al., 2018; Wrapp et al., 2020). There is a second cleavage site, S2′, located 130 residues from the N terminus of the S2 subunit that is highly conserved among coronaviruses. Cleavage at the S2′  site by host cell proteases is vital for successful infection by coronaviruses (Belouzard, Chu & Whittaker, 2009; Gui et al., 2017; Park et al., 2016; Walls et al., 2017).

The surface-exposed S glycoprotein is the main focus for therapeutic design and vaccine development as it is the main target of nAbs upon infection. The post-fusion S glycoprotein is strategically decorated with N-linked glycans, which have been suggested as a potential viral protective strategy to evade the host immune response (Cai et al., 2020). Moreover, N-linked glycans are important in correct protein folding and for providing accessibility to host proteases and nAbs (Walls et al., 2016; Xiong et al., 2017). The full-length S glycoprotein, the S1 subunit and especially the RBD are capable of inducing T cell-mediated immune response as well as potent nAbs in individuals infected with MERS-CoV, SARS-CoV and SARS-CoV-2 (Amanat & Krammer, 2020; He et al., 2004; Lan et al., 2015; Suthar et al., 2020). Moreover, RBD-specific humoral and cellular immunity were detected in COVID-19 convalescent individuals, where significant concentrations of nAbs, IgG and IgM as well as T cell-specific interferon gamma (IFN-γ) were present (Ni et al., 2020). In a separate animal study, RBD-specific IgG accounted for half of the S-induced antibody responses, suggesting RBD is the dominant immunogen as the concentration of N-specific IgG induced was 30-fold lower (Gao et al., 2020). In addition, immunization with the recombinant RBD of SARS-CoV-2 has been shown to elicit a robust neutralizing antibody response, even in the absence of antibody-dependent enhancement (Quinlan et al., 2020). Therefore, RBD is a promising immunogen and has been widely used in vaccine development, providing protection against SARS-CoV-2.

Naturally, viruses accumulate mutations over time; thousands of mutations have been described, especially in the S glycoprotein. While majority of the mutations are disadvantageous to viral fitness, some confer selective advantages, such as increased transmissibility as well as decreased effectiveness of the host immune response. Mutations induce modifications in the S glycoprotein structures, causing a change in the antigenic properties. SARS-CoV-2 lineages B.1.1.7 (Alpha variant), B.1.351 (Beta variant), P.1 (Gamma variant), B.1.617.2 (Delta variant) and B.1.1.529 (Omicron variant) are the main variants of concern (VOC) not only because of their enhanced infectivity and transmissibility but also due to their abilities to potentially reduce neutralization by nAbs (Dejnirattisai et al., 2021; Salleh, Derrick & Deris, 2021; Wall et al., 2021; Wang et al., 2021a). These variants could limit the vaccine effectiveness, thus achieving herd immunity by vaccinations would therefore be more difficult.

### mRNA optimization

**Figure 2:**
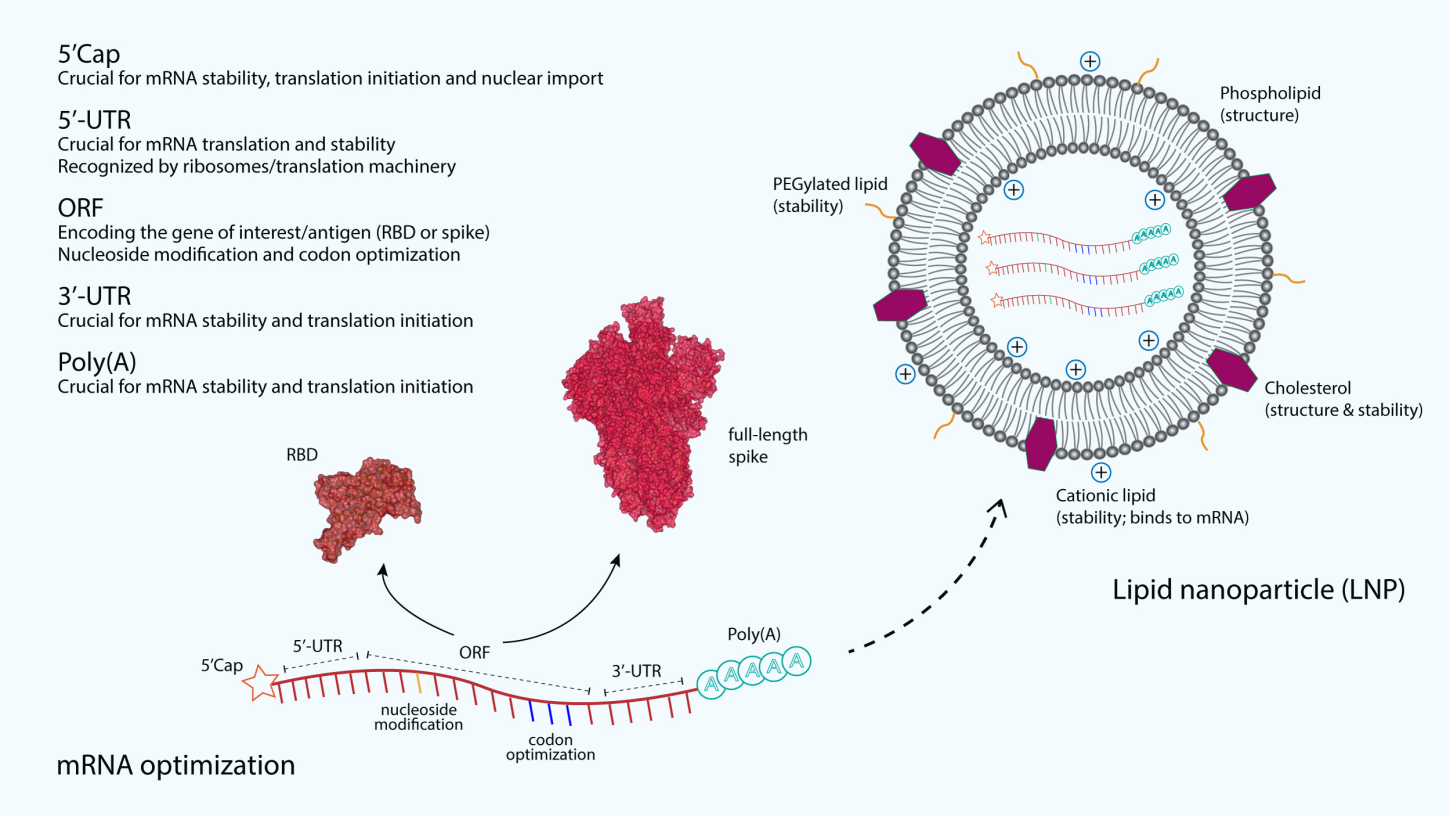
The optimization and delivery of mRNA vaccine. mRNA vaccines, developed against SARS-CoV-2, are optimized to include a 5′-capping and an untranslated region (UTR) at the 5′-end. The majority of mRNA vaccines developed against SARS-CoV-2 use a m7GpppNmN cap, also known as Cap 1, which is added to the 5′-end using the capping enzyme 2′-O-methyltransferase. These additional structures are added to increase the stability and improve the translation of mRNA. Similarly, an UTR and a poly(A) tail are added at the 3′-end of mRNA, both are crucial for the mRNA stability and translation initiation. The open reading frame (ORF), which encoding the receptor-binding domain (RBD) (PDB: 6M0J) (Lan et al., 2015) or the full-length spike (S) glycoprotein (PDB: 6VSB) (Wrapp et al., 2020), is optimized by using commonly occurring codons to replace rare codons. In addition, the sequence is modified to include GC rich content or modified nucleosides such as 1-methylpseudouridine. mRNA optimization by using modified nucleosides and common codons could potentially increase the immunostimulatory and efficacy of mRNA vaccines. In order to protect mRNA from the extracellular ribonucleases, amine-containing carrier molecules such as lipid nanoparticles (LNPs) are commonly used, which consist of phospholipid bilayer shell and ionizable cationic lipids. In addition, cholesterols and polyethylene glycol (PEG)-containing lipids are also added to increase the overall shell structure and stability.

The immunostimulatory capability of mRNA as well as its stability and translatability are the main critical concerns to be optimized in the mRNA vaccine development. Increased stability and translation can be achieved by optimizing many regions of the mRNA. For example, the 5′ and 3′ untranslated regions (UTRs) that are responsible for recruiting RNA-binding protein profoundly influence the stability and translation of mRNA (Fig. 2). These regulatory elements in both UTRs vastly increase the half-life and expression of mRNA (Holtkamp et al., 2006). Furthermore, the length of the 3′-UTR also plays an important role in increasing the translational efficiency and the stability of an mRNA, where the addition of 100 additional bases to the 3′-UTR has been shown to increase the mRNA translational efficiency and stability in Chinese hamster ovary cells by 38-fold and 2.5-fold, respectively (Tanguay & Gallie, 1996). In addition, poly(A) tail also plays a key regulatory role in the mRNA stability and translational efficiency. Polyadenylation can be added by using poly(A) polymerase or directly from the encoding DNA template. An optimal length of poly(A) tail stabilizes mRNA and increases protein translation (Pardi et al., 2018). The Moderna mRNA-1273 and Pfizer BNT162b2 vaccines are produced using a sequence-optimized mRNA encoding the SARS-CoV-2 S protein, flanked by 5′-UTR and 3′-UTR sequences and a poly(A) tail (Corbett et al., 2020a; Vogel et al., 2021).

The addition of 5′ Cap structure by using various versions of synthetic cap analogues is crucial for efficient protein translation. For instance, the translational efficiency of ‘anti-reverse’ cap analogue-capped mRNAs has been greatly increased as the conventional reverse cap structures reduce the translational efficiency of mRNAs (Grudzien-Nogalska et al., 2007; Stepinski et al., 2001). Apart from improved translation, 5′-capping is also important for facilitating pre-mRNA splicing, providing protection for mRNA from exonuclease degradation and acting as the binding site for the translation initiation complex eIF4F (Park et al., 2021). For majority of mRNA vaccines developed for SARS-CoV-2, including the licensed Pfizer and Moderna vaccines, a m7GpppNmN cap, also known as Cap 1 was added to the 5′-end using capping enzyme or 2′-O-methyltransferase (Corbett et al., 2020a; De Alwis et al., 2021; McKay et al., 2020; Sahin et al., 2020). Moreover, codon optimization by modifying rare codons in protein-coding sequences with identical commonly occurring codons can result in improvements in protein expression levels. The reason for this is because commonly preferred codons correlate with the abundance of cognate tRNAs that are available in the cytoplasm (Gustafsson, Govindarajan & Minshull, 2004; Hanson & Coller, 2017). Another example is that the enrichment of G:C content in the mRNA sequence. The translational efficiency of a GC-rich sequence has been shown to be 100-fold higher than that of a GC-poor counterpart (Kudla et al., 2006). Although these codon optimizations could positively modulate the protein expression, it is also possible that these changes could also affect mRNA secondary structure conformation, the kinetics of translation as well as its subsequent protein folding, which then could potentially influence the specificity and magnitude of the immune response (Buhr et al., 2016; Pardi et al., 2018; Yu et al., 2015).

Past studies have shown that the immunostimulatory ability of mRNA can be influenced by the introduction of modified nucleosides aside from complexing mRNA with various carrier molecules, which we have discussed above. Incorporation of naturally modified nucleosides, such as m5C, m6A, m5U, s2U or pseudouridine and 1-methylpseudouridine into mRNA prevents activation of the endosomal sensors Toll-like receptor 3 (TLR-3), TLR-7 and TLR-8 (Nance & Meier, 2021). RNA recognition by these endosomal sensors induces type I interferon (IFN) production and thus incorporating modified nucleosides could potentially reduce type I IFN signaling, hence decreasing the host innate immune response towards the RNA (Karikó et al., 2005). Nucleoside modifications also partially suppress the recognition of double-stranded (ds)RNA species, resulting in more efficient translation of nucleoside-modified mRNA, compared to unmodified mRNA *in vitro*, especially in primary dendritic cells (DCs) and *in vivo* in mice, where the highest level of protein production was observed (Pardi et al., 2018). Conversely, the immunostimulatory properties of mRNA can also be increased by the introduction of an adjuvant (Pardi et al., 2018). Although the information on the use of adjuvant in mRNA vaccines against SARS-CoV-2 is scant, there are few inactivated, subunit and virus-like particle vaccines against SARS-CoV-2 have been developed with adjuvants. The use of adjuvants in coronavirus vaccines has been extensively reviewed (Liang et al., 2020). Adjuvants such as CpG 1018, AS03 and MF59 have been licensed to be used in vaccines against SARS-CoV-2 by Dynavax, GlaxoSmithKline and Seqirus, respectively (Liang et al., 2020; Thanh Le et al., 2020). AS03 and MF59 are emulsion adjuvants, which have been previously used in vaccines developed against SARS-CoV and MERS-CoV. AS03 elicited both potent humoral and cellular immune responses to an inactivated SARS-CoV vaccines, where the use of the adjuvant resulted in higher antibody titers compared to non-adjuvanted vaccines (Roberts et al., 2010). Furthermore, self-amplifying mRNA vaccines that have been formulated with a cationic nanoemulsion adjuvant based on the licensed MF59, have shown an increase in immunogenicity and potency in a variety of animal models (Brito et al., 2014). Apart from emulsion adjuvants, salt-based aluminum adjuvants such as aluminum hydroxide were also used. They were the first adjuvants used in licensed human vaccines and are still commonly used due to their safety and ability to increase immune responses (Liang et al., 2020). A recent study of the inactivated SARS-CoV-2 vaccine, CoronaVac, which is adjuvanted with aluminum hydroxide provided complete protection in non-human primates with potent humoral response (Gao et al., 2020).

### Lipid nanoparticle

mRNA vaccines require an efficient delivery for their success and clinical translation in the cytoplasm. mRNA vaccines are designed to be administered with a carrier molecule that protects the genetic material from degradation by omnipresent extracellular ribonucleases and delivers it to the cytoplasm without substantial toxicity. To reach the cytoplasm, the mRNA must cross the cell membrane, a dynamic and formidable barrier, in which both mRNA molecule and the cell membrane are negatively charged. Recent advances in the design of mRNA manufacturing and intracellular delivery methods have vastly improved the mRNA delivery system. Multiple types of polymers, peptides and lipid-based carriers have shown promise in preclinical and some clinical studies (Kowalski et al., 2019). In order to protect mRNA against ribonuclease degradation and to shield its negative polarity, amine-containing carrier molecules such as lipid nanoparticles (LNPs) are commonly used for *in vivo* mRNA delivery among potential non-viral vectors (Fig. 2). LNPs can be manufactured in large-scale with relative ease, and low-cost. LNPs can be co-formulated with adjuvants and surface-decorated with ligands, receptors and antigens to facilitate the uptake by the desired type of immune cells, such as antigen-presenting cells (APCs). For example, coating the particles with a polyethylene glycol (PEG)-containing lipid can reduce complement system activation facilitating longer presence in the circulation, thus improving the vaccine delivery (Reichmuth et al., 2016).

**Figure 3:**
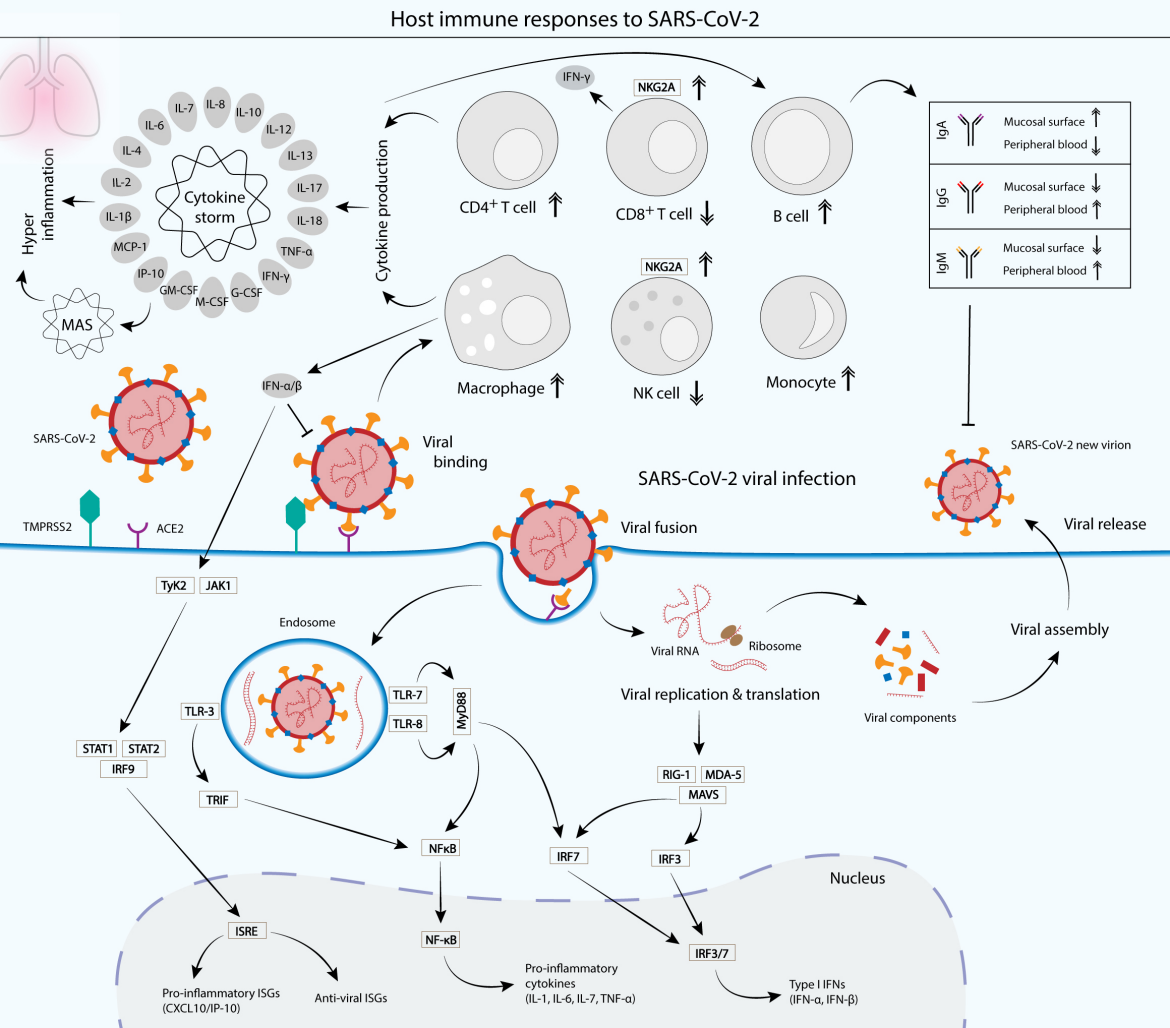
Cellular and humoral host immune responses to SARS-CoV-2 viral infection. SARS-CoV-2 viral entry into the epithelial host cells is mediated by the S glycoprotein binding to the cell receptor angiotensin-converting enzyme (ACE2) via the RBD of the S1 subunit. The binding depends on the type II transmembrane serine protease (TMPRSS2) for the S protein priming (Hoffmann et al., 2020) . Upon entry, the virus is recognized by the innate immune receptors, such as toll-like receptor 3 (TLR-3), TLR-7/8 and RIG-1/MDA-5, leading to the subsequent production of type I interferons (IFNs) and pro-inflammatory cytokines (interleukin 1 (IL-1), IL-6, IL-7 and tumor necrosis factor alpha (TNF-α)). Type I IFNs such as IFN-α and IFN-β are crucial in suppressing the propagation of the virus and essential in the activation of pro-inflammatory and anti-viral IFN-stimulated genes (ISGs) via the JAK-STAT signaling pathway (McNab et al., 2015) . Cytokines produced by infected cells then modulate the adaptive immune response via the activation of a plethora of immune cells such as macrophages, natural killer (NK) cells, CD4^+^ T cells and B cells, which produce IgA, IgG and IgM antibodies to neutralize the virus (Chen et al., 2020a; DelValle et al., 2020; Ni et al., 2020). However, an unbalanced immune response that leads to hyperactivation of the inflammatory immune response causes a phenomenon called cytokine storm and its associated macrophage activation syndrome (MAS), which leads to immune exhaustion and causes severe clinical symptoms of coronavirus disease 2019 (COVID-19) (Song et al., 2020). Patients with SARS-CoV-2 infection displayed a functional exhaustion of NK and CD8^+^ T cells, associated with an increased expression of the inhibitory receptor NKG2A (Antonioli et al., 2020; Zheng et al., 2020).

Adjuvants, conversely can be added to LNPs to increase the immune response (Reichmuth et al., 2016). Adjuvants such as 1,2-dioleyl-3-trimethylammonium-propane chloride salt (a cationic lipid), have been shown to activate TLR-4, which in turn increasing the production of T helper cytokines, such as interleukin-2 (IL-2), tumor necrosis factor alpha (TNF-α) and IFN-γ (Kedmi, Ben-Arie & Peer, 2010). Aluminum salts were first utilized to increase the immune response of conventional vaccines. It is used in inactivated vaccines against SARS-CoV-2 such as CoronaVac and BBIBP-CorV, which contain aluminum hydroxide as adjuvants (Gao et al., 2020; Wang et al., 2020). However, mRNA vaccines against SARS-CoV-2 do not require the use of other adjuvants as the LNP seems to serve as an adjuvant (Chung et al., 2020). Some lipids have been reported to have an adjuvant effect by themselves and can activate the immune response and induce inflammation (Reichmuth et al., 2016). Although Pfizer/BioNTech and Moderna do not explicitly mention the use of an adjuvant, but mRNA already contains immunostimulatory properties through the activation of pathogen recognition receptors (Chung et al., 2020). Previous studies have shown that mRNA vaccines against influenza demonstrated potent self-adjuvantation and induced balanced immune responses comprising both humoral and cellular effector responses. mRNA poses self-adjuvantation via its recognition by the endosomal sensors TLR-3, TLR-7 and TLR8, and the cytoplasmic sensors such as RIG-I or MDA-5 (Fig. 3) (Edwards et al., 2017; Kowalczyk et al., 2016).

LNP generally consists of a lipid bilayer shell that surrounds an aqueous core and is usually made of a combination of different kinds of lipids, each has its own distinct functions (Fig. 2). LNP formulations mostly rely on ionizable cationic lipids, whose positive charge efficiently binds to the negatively charged RNA. Some anionic and neutral formulations, such as phospholipids and cholesterol are used for their structural roles in LNP. Phospholipids facilitate endosomal escape by disrupting the lipid bilayer and promote a transition from a lamellar to a hexagonal phase in the endosome (Cui, Chen & Zhu, 2008; Reichmuth et al., 2016). Cholesterol serves as a stabilizing agent in LNP and is also important in promoting the transition from lamellar to hexagonal phases. The hexagonal phase is crucial for dissociation of mRNA from LNP and its translocation across the endosomal membrane into the cytosol (Zuhorn et al., 2005). Cholesterol has been shown to strengthen fluid bilayers and reduce the leakage of contents from liposomes (Allen & Cullis, 2013). Lipid-anchored PEGs are formulated on the LNP surface, where they serve as a barrier that stabilizes LNP and reduces nonspecific binding to proteins. They also reduce cellular uptake by the reticulo-endothelial system and interaction with the endosomal membrane, while increasing the blood circulation time of LNP in order to reach target immune cells such as APCs (Reichmuth et al., 2016).

## COVID-19 and Immune Response

SARS-CoV-2 infects mammalian lung epithelial cells by binding via its S glycoprotein to the ACE2 receptor, which is abundantly expressed on the surface of the lung and intestine, and less frequently, in the kidney, heart and adipose tissues (Murgolo et al., 2021). Binding to the receptor activates proteolytic cleavage by the type II transmembrane serine protease (TMPRSS2) near the junction between the S1 and S2 subunits, which leads to viral and host cellular membrane fusion, and subsequent transfer of the viral RNA into the host cell cytoplasm (Fig. 3) (Hoffmann et al., 2020). Infection with SARS-CoV-2 induces both humoral and cell-mediated immunity in humans (Chen et al., 2020a; Del Valle et al., 2020; Ni et al., 2020). Various innate recognition and response cascades are triggered by the SARS-CoV-2 infection that are crucial for the recognition and suppression of pathogens, and for subsequent activation of the adaptive immune system. Since the lung epithelial cells serve as the main tissue tropism for SARS-CoV-2 infection, the mucosal surfaces present the first line of defense. A previous study has reported that early SARS-CoV-2-specific humoral responses were dominated by IgA antibodies, with high concentrations detected in the saliva and bronchoalveolar lavage fluid, within three weeks upon infection (Sterlin et al., 2021). Antibody responses against SARS-CoV-2 include IgA, IgG and IgM, but IgA has been reported to contribute to the virus neutralization at a greater extent compared with IgG (Sterlin et al., 2021). Moreover, dimeric IgA antibodies that are abundantly found in mucosal tissues including the upper respiratory tract, neutralized SARS-CoV-2 15 times more potently than their monomeric counterparts (Wang et al., 2021c). Blood samples collected from convalescent individuals reported that N- and RBD-specified IgG and IgM antibodies were both detected in the blood sera, significantly at high levels even after at least 2 weeks after discharge (Ni et al., 2020). This demonstrates that natural infection with SARS-CoV-2 induces a robust humoral response, which lasts weeks after the infection.

Cytokines such as IFNs and ILs play important roles in the immune response against SARS-CoV-2. Pro-inflammatory cytokines such as IL-1β, IL-6, IL-12, IL-17, IFN-γ and TNF-α, as well as cytokines involved in anti-inflammation like IL-10 have also been described to be elevated in COVID-19 patients. In addition, various studies have also described abnormal levels of cytokines that are involved in adaptive immunity such as IL-2 and IL-4 in the patients. Moreover, other cytokines that are directly or indirectly involved in the activation of immune cascades, which serve as important regulators of immune responses such as IL-7, IL-13, M-CSF, G-CSF, GM-CSF, IP-10 and MCP-1 have also been observed to be elevated in the COVID-19 patients (Chen et al., 2020a; Costela-Ruiz et al., 2020; Huang et al., 2020). It has been shown that hyper activation of the inflammatory immune response can lead to a phenomenon called cytokine storm (Fig. 3) and subsequent immune exhaustion which may lead to disease severity (Song et al., 2020). For instance, excessive production of the pro-inflammatory cytokines TNF-α and IFN-γ synergistically induce inflammatory cell death, leading to tissue damage and eventually death (Karki et al., 2021). Furthermore, imbalanced and inappropriate inflammatory response, which is defined by low levels of type I and III IFNs contrasted to elevated chemokines and high expression of IL-6, drives the development of severe COVID-19 (Blanco-Melo et al., 2020). IL-6 is a crucial main factor in cytokine storm and has been reported to be present at high levels in severe COVID-19 patients, which may cause severe damage to lung tissue (Huang et al., 2020).

Infection with SARS-CoV-2 activates cell-mediated immunity, in which macrophages serve as one of the primary cellular defenses against the pathogen. Macrophages are a group of innate immune cells that can be found in the lungs and along the upper respiratory tract, where they are among the early immune cells to act on invading virions. They produce pro-inflammatory molecules such as IL-6 that destroy the threats. Their activities on the SARS-CoV-2 virus drive both inflammation and the progression of COVID-19. Severe infections, which induce macrophage activation syndrome (MAS) can lead to dysregulation of macrophage response that severely damage the host (Merad & Martin, 2020). MAS is associated with cytokine storm, with markedly increased production of pro-inflammatory cytokines such as TNF-α, IFN-γ, IL-1, IL-6, IL-7, and IL-18, as well as inflammatory chemokines including CXC-chemokine ligand 10 (CXCL10/IP-10), CC-chemokine ligand 2 (CCL2) and CCL3 (Merad & Martin, 2020; Schulert & Grom, 2015). Interestingly, several studies have shown that macrophages and monocytes can be infected by SARS-CoV-2 and the infection induced host immunoparalysis for the benefit of COVID-19 progression (Boumaza et al., 2021; Szekely et al., 2021). Monocytes are blood-circulating, phagocytic innate immune cells that play an important role in protecting the host organism against the invading pathogens (Knoll, Schultze & Schulte-Schrepping, 2021). However, a significantly higher percentage of CD14^+^CD16^+^ inflammatory monocytes was observed in the peripheral blood of COVID-19 patients admitted to intensive care units (ICUs), associated with markedly high expression of IL-6 as compared to non-ICU patients (Zhou et al., 2020). In contrast, a significant decrease of peripheral blood cytotoxic lymphocytes such as CD8^+^ T lymphocytes and natural killer (NK) cells is observed in severe COVID-19 patients. This functional exhaustion of cytotoxic lymphocytes is associated with increased expression of the inhibitory receptor NKG2A on the surface of NK and CD8^+^ T cells. More importantly, the number of NK and CD8^+^ T cells was elevated to normal levels with reduced expression of NKG2A in convalescent patients (Antonioli et al., 2020; Zheng et al., 2020).

## Activation of Adaptive Immune Response by mRNA Vaccines

### mRNA vaccine-induced antibody responses

Several studies showed that natural infection by SARS-CoV-2 induces potent nAb production in convalescent individuals. Thus, mRNA vaccines developed for COVID-19 have been focused on their ability to induce robust nAb responses. A strong, dose-dependent antibody response was observed in participants immunized with the Pfizer/BioNTech BNT162b1 vaccine. A previous report showed that RBD-specific IgG increased in a dose-dependent manner, with geometric mean concentrations (GMCs) in the range of 3,920–18,289 U ml^−1^ in BNT162b1-vaccinated individuals after 21 days of receiving a second dose (day 43), as compared to a GMC of 602 U ml^−1^ in non-vaccinated convalescent individuals. A second dose is necessary to elevate antibody concentrations as the IgG GMC of individuals who received a priming dose was only at 755 U ml^−1^ by day 43. Likewise, substantial increases in nAb titers were also observed in a dose-dependent manner after the second dose (Sahin et al., 2020). These results complement with the earlier Phase I/II study of the BNT162b1 vaccine, where a clear dose response was elicited with neutralizing GMTs observed after the second dose particularly, at higher dose concentrations (Mulligan et al., 2020). Similarly, a safety and immunogenicity report of another Pfizer/BioNTech vaccine candidate BNT162b2, which is currently licensed under the brand name Comirnaty, showed that the vaccine elicited similar dose-dependent SARS-CoV-2-neutralizing GMTs, which were similar to or higher than the GMT of a panel of SARS-CoV-2 convalescent serum samples. IgG and virus-neutralizing responses to vaccination with 10 µg to 30 µg of BNT162b2 were boosted by the second dose, reaching highest neutralization titers on day 35 (two weeks after the second dose), which was similar to vaccination with BNT162b1 (Walsh et al., 2020). Although BNT162b2 is quite effective in providing protection against the wild-type SARS-CoV-2 infection, there is a time-dependent reduction in nAbs has been reported, which was associated with lower nAb activity against SARS-CoV-2 variants such as Delta (Tartof et al., 2021). This has prompted the recommendation of a third dose, particularly after the emergence of the Omicron variant. Several reports showed that the third dose of BNT162b2 provided added protection against SARS-CoV-2 (Peled et al., 2021; Saciuk et al., 2022; Terpos et al., 2022). The third dose elicited neutralization titers and IgG anti-RBD antibodies of more than 9-fold and 3-fold higher, respectively than the range achieved after the second dose (Peled et al., 2021).

Immunogenicity evaluation of the mRNA-1273 vaccine, which is currently sold under the brand name Spikevax, in non-human rhesus macaques receiving 10 or 100 µg of the vaccine showed a robust neutralizing activity and rapid protection in the upper and lower airways of the primates. The vaccine induced neutralizing GMTs of 501 and as high as 3481 in the 10- and 100-µg dose groups, respectively. In addition, S-specified IgG antibody response was markedly increased in a dose-dependent manner after two vaccinations (Corbett et al., 2020a). In another study, the mRNA-1273 vaccine has been shown to induce potent neutralizing responses to both wild-type SARS-CoV-2 and D614G mutant, which has become dominant around the world (Corbett et al., 2020a). Similar to BNT162b2, it has been reported that there was a marked decrease in antibody after 6 months of vaccination with mRNA-1273 (Tré-Hardy et al., 2021). The third dose has been shown to increase nAb response against the SARS-CoV-2 variants such as Alpha, Beta and Delta, compared to the wild-type (Kumar et al., 2021).

Safety and immunogenicity assessment of another mRNA vaccine, CVnCoV, developed by CureVac showed robust immune responses in all groups of participants, especially of the S- and RBD-specified IgG production (Kremsner et al., 2021). Although the vaccine has been proven to induce strong humoral responses with high titers of virus-nAbs in its Phase I clinical (Kremsner et al., 2021) as well as previous pre-clinical studies in mice and hamsters (Hoffmann et al., 2021a; Rauch et al., 2021), its Phase II/III clinical trial showed the CVnCoV vaccine’s efficacy against symptomatic COVID-19 is only 48% (CureVac, 2021). Moreover, nAb levels in the recipients who received the CVnCoV vaccine were comparable with those in sera from convalescent patients (Kremsner et al., 2021), but much lower than those recipients who received the Pfizer and Moderna vaccines, possibly due to lower 12 µg dose used, as compared to 30 µg and 100 µg for the Pfizer and Moderna vaccines, respectively. Due to low efficacy recorded from its clinical trial, CureVac has totally abandoned the development of CVnCoV and now has moved forward in developing a second-generation mRNA vaccine, in collaboration with GlaxoSmithKline (Adams, 2021).

### Activation of T cells and cytokine production

**Figure 4:**
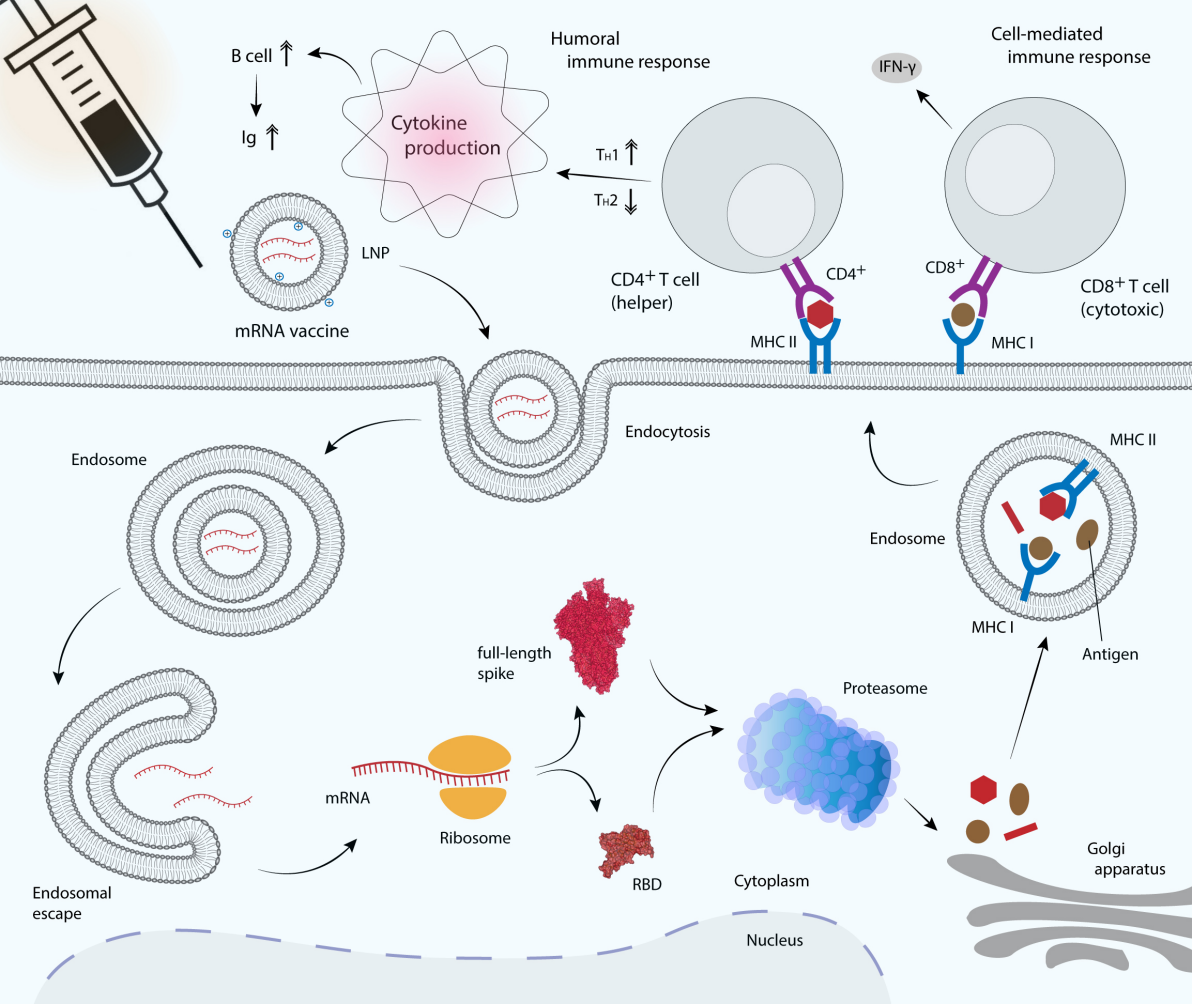
mRNA vaccine delivery by LNP and its mechanism in promoting the adaptive immune response. mRNA vaccines are delivered to antigen presenting cells such as dendritic cells, macrophages and B cells. LNP protects mRNA from enzymatic degradation by extracellular ribonucleases. Positively charged ionizable cationic lipids favor mRNA localization at the negatively charged cell membrane, as well as facilitate endocytosis and the subsequent endosomal escape (Reichmuth et al., 2016). Upon translation, the RBD or full-length S glycoprotein will be secreted out of the host cell or processed into smaller peptides by proteasome. Processed antigenic peptides are transported into the endoplasmic reticulum (not shown) and Golgi apparatus, and loaded onto major histocompatibility complex (MHC) class I or class II molecules, where they will be subsequently presented on the cell surface. MHC I is recognized by the cytotoxic T cell via CD8^+^ receptor, whereas MHC II is recognized by the helper T cell via CD4^+^ receptor, leading to the production of inflammatory cytokines and the subsequent induction of humoral and cell-mediated immune responses.

Infection by SARS-CoV-2 stimulates the production of nAbs, virus-specific T cells as well as robust immune-modulatory cytokines. As a key factor for several antiviral responses, IFN-γ has been shown to act synergistically with IFN-β in inhibiting SARS-CoV replication (Sainz et al., 2004). The Phase I/II clinical trial of the Pfizer BNT162b1 vaccine showed that IFN-γ and IL-2 were secreted primarily by the RBD-specific CD4^+^ T cells and fractions of CD8^+^ T cells, in agreement with the previous preclinical studies in mice and macaques (Sahin et al., 2020; Vogel et al., 2021). BNT162b vaccines elicited high level of specific CD4^+^ T cells that primarily produced the T-helper-1 (T_H_1) cytokines IFN-γ, IL-2 or TNF-α, as opposed to the T-helper-2 (T_H_2) cytokines IL-4, IL-5 or IL-13 (Fig. 4), indicating a T_H_1-biased response (Sahin et al., 2020; Vogel et al., 2021). Increase in the strength of CD4^+^ T cell responses has most likely contributed to the high RBD-binding IgG and nAb titers. Although the strength of CD8^+^ T cell responses correlated positively with CD4^+^ T cell responses, it did not significantly correlate with nAb titers. Interestingly, unlike RBD-specific IgG and nAb responses, T cell responses were not dose-dependent, in which a dose as low as 1 µg has been shown to elicit a robust expansion of T cells in most patients (Sahin et al., 2020). However, in a separate study using the mRNA-1273 vaccine showed that T_H_1 response is dose-dependent and the response levels were higher in the 100-µg dose compared to the control or in the 10-µg dose groups. T_H_2 responses, conversely remained low or undetectable in both 10- and 100-µg dose groups. CD40L, a cell–surface marker that is abundantly expressed after CD4^+^ T-cell activation and IL-21, which is produced by CD4^+^ T follicular helper (TfH) cells were also detected in both groups. CD40L is important for B-cell activation and efficient isotype switching whereas TfH cells are crucial for generation of long-term B-cell memory (Corbett et al., 2020b).

Vaccines provide long-term protection from infection by generating memory B cells and long-lived plasma cells, which persistently produce antigen-specific antibodies. Memory B cells that produce full-length S protein- and RBD-specific IgG1 and IgG2a/b were detected at significantly high concentrations, along with full-length S protein- and RBD-specific IgM-producing B cells, after administration of a single vaccine dose (Laczkó et al., 2020). Activation of T cell response depends on the number of antigenic determinants, in which the full-length S protein vaccine has been shown to induce greater T cell responses compared to the shorter RBD vaccine. This is because the longer full-length S protein produced by mRNA vaccines contains additional T cell epitopes and therefore, the vast majority of CD4^+^ T cells recognize epitopes in both the N-terminal and C-terminal domains of the S protein, while interestingly the majority of CD8^+^ T cell response was directed only at epitopes in the N-terminal domain (Laczkó et al., 2020). CVnCoV, which encodes the full-length SARS-CoV-2 S protein has been shown to induce both S-specific CD4^+^ and CD8^+^ T-cell responses in mice (Rauch et al., 2021). Moreover, immunization with the S1 domain induced much higher IgG and IgA antibody levels than the RBD vaccine (Wang et al., 2021d). In contrast, vaccination with mRNA-1273 that encoding the full-length prefusion-stabilized SARS-CoV-2 S-2P protein has been shown to induce low or undetectable CD8^+^ T cell response, although the CD4^+^ T cell response remained high (Corbett et al., 2020b). However, in a later study, mRNA-1273 has been shown to induce a robust CD8^+^ T cell response, with a balanced T_H_1/T_H_2 response (Corbett et al., 2020a). In addition, immunization with two doses of BNT162b1 that encoding the trimerized RBD elicited robust CD4^+^ and CD8^+^ T cell responses and strong antibody responses, with RBD-binding IgG concentrations higher than those observed in sera from convalescent individuals (Sahin et al., 2020). High CD8^+^ T cells detected were possibly due to RBD multimerization, a strategy used to optimize its immunogenicity.

## Challenges and Limitations of COVID-19 mRNA Vaccines

mRNA vaccine represents a promising alternative compared to conventional vaccines due to their high potency and ability for rapid development, with cost-efficient production. However, the physiochemical properties of mRNA may influence its stability, cellular delivery and organ distribution. Thus, optimal construction of mRNA for the delivery and translation is required to reduce the risks of degradation, posed by extracellular RNases. The use of molecular carrier that was discussed above, such as LNPs, co-formulated with adjuvants and surface-decorated with ligands, receptors and antigens would facilitate the cellular uptake of mRNA vaccines and reduce degradation. mRNA needs to be engineered to resemble fully processed mature mRNA molecules as they occur naturally in the cytoplasm of eukaryotic cells. The mRNA products should contain an open reading frame (ORF) that encodes the protein of interest, flanking UTRs, a 5′ Cap and a poly(A) tail (Pardi et al., 2018). These modifications could not only provide extra protection from RNases, but are also important for facilitating and improving protein translation (Holtkamp et al., 2006; Pardi et al., 2018; Park et al., 2021). mRNA optimization by using modified nucleosides and common codons could also increase the immunostimulatory capability and efficacy of mRNA vaccines. For instance, the Pfizer BNT162b2 and Moderna mRNA-1273 vaccines, which use uracil-modified mRNA have been proven to give 95% efficacy in preventing COVID-19 (Baden et al., 2021; Polack et al., 2020). However, CVnCoV, the unmodified mRNA vaccine with naturally occurring nucleotides encoding the full-length S protein failed to provide the minimum efficacy in Phase III trials with only 48% protection against COVID-19 (CureVac, 2021; Kremsner et al., 2021). This is because the unmodified mRNA that is detected by the endosomal sensors TLR-7 and TLR-8 may trigger the production of type I IFNs, which can block the activation of B cells and subsequent production of antibodies (Karikó et al., 2005; McNab et al., 2015; Pardi et al., 2018).

The CD4^+^ T cell activation is critical for the activation of B cells and antibody production. However, the induction of T_H_2 cytokines such as IL-4, IL-5 and IL-13 has been associated with vaccine-associated enhanced respiratory disease (VAERD), a set of diseases with predominant involvement of the lower respiratory tract. VAERD was previously observed in some preclinical animal models of SARS and MERS coronavirus vaccines (Bolles et al., 2011). Examples include enhanced respiratory syncytial virus infection and atypical measles, occurring after administration of vaccines for the respective viruses (Munoz et al., 2021). Therefore, it is imperative to achieve a balance T_H_1/T_H_2 cytokine production as high COVID-19-associated mortality has been observed from an imbalance T_H_1/T_H_2 response (Pavel et al., 2021). For instance, VAERD is usually not observed when a CD4^+^ T_H_1 response occurs in the absence of a T_H_2 response. The Ig subclass and T cell cytokine data from mRNA-1273 shows that the immunization with the vaccine elicited a balanced T_H_1/T_H_2 response, in which low IgG2a/IgG1 ratios and low levels of IL-4, IL-5 and IL-13 were recorded (Corbett et al., 2020a). In addition, low or undetectable levels of the T_H_2 cytokines IL-4, IL-5 or IL-13 were observed after the immunization with the BNT162b vaccines, whereas strong T_H_1-type CD4^+^ T cell responses were recorded (Vogel et al., 2021). All these data together demonstrate that the mRNA vaccines, which are currently deployed against SARS-CoV-2 are capable in avoiding T_H_2-biased immune responses that have been linked to VAERD.

There are few rare complications associated with mRNA vaccinations, especially in young adults and adolescent males. Several vaccine-associated cases of myocarditis, pericarditis and cardiomyopathy have been reported, which occurred within a week after receiving the second dose of either the Pfizer BNT162b2 or Moderna mRNA-1273 vaccines (Bozkurt, Kamat & Hotez, 2021; Kim et al., 2021; King et al., 2021; Pepe, Gregory & Denniss, 2021). Acute myocarditis is most commonly due to viral infections but rare cases of vaccine-associated myocarditis have previously been reported in healthy adults that received the smallpox and influenza vaccines (Cheng et al., 2016; Kuntz et al., 2018). Although the exact mechanisms for myocarditis are obscure, several potential mechanisms of myocarditis linked to mRNA vaccinations have been proposed. In certain individuals with genetic predisposition, the immune system may detect the mRNA molecule as an antigen, thus activating pro-inflammatory cascades and immunologic pathways, leading to dysregulated cytokine expression (Bozkurt, Kamat & Hotez, 2021). In addition, nAbs against the SARS-CoV-2 S glycoprotein have been shown to cross-react with structurally similar human proteins such as α-myosin and transglutaminases, possibly leading to an increase in autoimmune diseases (Vojdani & Kharrazian, 2020). Further evaluation of these rare complications upon vaccination with mRNA vaccines is warranted to assess potential risk of morbidity, including cardiac injury.

Since the emergence of SARS-CoV-2 in the late 2019, over a thousand mutations have been recorded and some confer resistance to the virus. Certain combinations of specific point mutations that induce modifications in the S glycoprotein structures, leading to a change in the antigenic properties proving to be more alarming than others. VOCs such as Alpha, Beta, Gamma, Delta and Omicron variants are concerning because of their increased transmission and they can potentially decrease neutralization by nAbs, which may render currently deployed vaccines less effective (Salleh, Derrick & Deris, 2021). For instance, N501Y mutation, which is present in the Alpha, Beta and Gamma variants is found in the RBD and has been shown to confer resistance by disrupting antibody binding (Supasa et al., 2021). Sera obtained from the individuals who have been immunized with the mRNA-1273 and BNT162b2 vaccines showed a moderate 2-fold and 3.3-fold reduction, respectively in the neutralization against the Alpha variant (Shen et al., 2021; Supasa et al., 2021). Moreover, neutralization of the Gamma variant by the BNT162b2 vaccine showed a reduction of 2.6-fold, relative to the original wild-type strain, significantly better than the neutralization of the Beta variant, which recorded a 7.6-fold reduction (Dejnirattisai et al., 2021). Although these findings have showed reductions in neutralization, vaccine efficacy remains high and for now, these variants are not major concerns for the current COVID-19 vaccines. Moreover, vaccine-elicited CD4^+^ T cells have been shown to effectively recognize some of the VOCs, providing protection from severe disease even in the absence of effective nAbs (Woldemeskel, Garliss & Blankson, 2021). However, new emerging mutations would further challenge the vaccine efficacy and therefore, it is imperative to continuously assess these vaccine efficacies against potential emerging new variants. For instance, the E484K mutation that was previously found in the Beta and Gamma variants, has been recently discovered in the Alpha variant. The mutation has been shown to further reduce antibody neutralization, by disrupting the binding of serum polyclonal nAbs (Jangra et al., 2021). The recent emergence of the Omicron variant, which has been recently designated as a VOC on 26th November 2021 is alarming as it harbors a large number of mutations, particularly 30 mutations in the S glycoprotein, half of which are located in the RBD (Araf et al., 2022). Some of its S mutations such as K417N, T478K, N501Y and D614G, all of which are also found in the previous VOCs, are responsible in enhanced viral transmission and immune evasion (Salleh, Derrick & Deris, 2021; Shanmugaraj et al., 2021). Furthermore, H655Y and N679K mutations, both are located near the furin cleavage site and predicted to increase S cleavage, thus enhancing the viral transmissibility (Peacock et al., 2021). Nevertheless, whether the current COVID-19 vaccines could potentially resist the new Omicron variant is still under investigation. However, several recent reports suggested that the Omicron variant is more likely to evade neutralization by antibodies from individuals vaccinated with the Pfizer/BioNTech vaccine, compared to the wild-type and Delta variant (Hoffmann et al., 2021b; Lu et al., 2021). It is concerning that emerging variants of SARS-CoV-2, through mutations in the S glycoprotein, especially the RBD can evade nAbs induced by vaccination. Furthermore, it has been reported that there is a time-dependent reduction in nAbs of mRNA vaccines, which was associated with lower nAb activity against SARS-CoV-2 variants such as Delta (Tartof et al., 2021) that has prompted the administration of a third dose, particularly after the emergence of the Omicron variant. Although the third dose has been shown to elicit high and robust immune responses against the current VOCs (Peled et al., 2021; Saciuk et al., 2022; Terpos et al., 2022), if the immunity continues to wane over the time for mRNA vaccines, it is possible more doses will be required to confer prolong protection against newly emerging variants, and to provide durable and sustainable immunity against SARS-CoV-2. Therefore, urgent safety and efficacy assessment against newly emerging variants is required to verify the effectiveness of these vaccines.

## Conclusion

mRNA vaccines have emerged as promising alternatives to provide protection against SARS-CoV-2 due to their high potency with the capacity for rapid development and low-cost production. Immunization with two doses of mRNA vaccine elicited a robust CD8^+^T cell response, with a balanced CD4^+^ T_H_1/T_H_2 response. High levels of S-specific IgG and nAbs were also detected. mRNA vaccines have been shown to provide a robust protection with high efficacy against the virus, newly emerging variants of SARS-CoV-2 may render the currently deployed mRNA vaccines less effective. Although these challenges and limitations as well as adverse events following immunization pose potential risks to the development of the mRNA vaccines, their benefits in providing protection against SARS-CoV-2 outweigh the risks.

## Additional Information and Declarations

### Data Availability

The following information was supplied regarding data availability: This is a literature review.
